# Neuroendocrine Humoral and Vascular Components in the Pressor Pathway for Brain Angiotensin II: A New Axis in Long Term Blood Pressure Control

**DOI:** 10.1371/journal.pone.0108916

**Published:** 2014-10-02

**Authors:** John M. Hamlyn, Cristina I. Linde, Junjie Gao, Bing S. Huang, Vera A. Golovina, Mordecai P. Blaustein, Frans H. H. Leenen

**Affiliations:** 1 Department of Physiology, University of Maryland Baltimore, Baltimore, Maryland, United States of America; 2 Department of Medicine, University of Maryland Baltimore, Baltimore, Maryland, United States of America; 3 University of Ottawa Heart Institute, Ottawa, Ontario, Canada; School of Pharmacy, Texas Tech University HSC, United States of America

## Abstract

Central nervous system (CNS) administration of angiotensin II (Ang II) raises blood pressure (BP). The rise in BP reflects increased sympathetic outflow and a slower neuromodulatory pressor mechanism mediated by CNS mineralocorticoid receptors (MR). We investigated the hypothesis that the sustained phase of hypertension is associated also with elevated circulating levels of endogenous ouabain (EO), and chronic stimulation of arterial calcium transport proteins including the sodium-calcium exchanger (NCX1), the type 6 canonical transient receptor potential protein (TRPC6), and the sarcoplasmic reticulum calcium ATPase (SERCA2). Wistar rats received a chronic intra-cerebroventricular infusion of vehicle (C) or Ang II (A, 2.5 ng/min, for 14 days) alone or combined with the MR blocker, eplerenone (A+E, 5 µg/day), or the aldosterone synthase inhibitor, FAD286 (A+F, 25 µg/day). Conscious mean BP increased (P<0.05) in A (123±4 mm Hg) vs all other groups. Blood, pituitary and adrenal samples were taken for EO radioimmunoassay (RIA), and aortas for NCX1, TRPC6 and SERCA2 immunoblotting. Central infusion of Ang II raised plasma EO (0.58±0.08 vs C 0.34±0.07 nM (P<0.05), but not in A + E and A + F groups as confirmed by off-line liquid chromatography (LC)-RIA and LC-multistage mass spectrometry. Two novel isomers of EO were elevated by Ang II; the second less polar isomer increased >50-fold in the A+F group. Central Ang II increased arterial expression of NCX1, TRPC6 and SERCA2 (2.6, 1.75 and 3.7-fold, respectively; P<0.01)) but not when co-infused with E or F. Adrenal and pituitary EO were unchanged. We conclude that brain Ang II activates a CNS-humoral axis involving plasma EO. The elevated EO reprograms peripheral ion transport pathways known to control arterial Na^+^ and Ca^2+^ homeostasis; this increases contractility and augments sympathetic effects. The new axis likely contributes to the chronic pressor effect of brain Ang II.

## Introduction

The central nervous system (CNS) plays a significant role in human essential hypertension and in many experimental models of hypertension [1], [2]. The CNS influences BP via peripheral sympathetic nerve activity (SNA) and via long-range endocrine mechanisms including adrenocorticotropic hormone (ACTH), growth hormone, angiotensin II (Ang II) and vasopressin [2]–[5]. Circulating Ang II can enter the CNS via fenestrated epithelia in the hypothalamus and also is generated within the brain [6]–[8]. Increases in central Ang II raise sympathetic nerve activity, elevate the plasma levels of vasopressin and ACTH and increase BP [9]. The observation that the prolonged peripheral administration of low, sub-pressor doses of Ang II elevates BP gradually [10]–[12] led to the idea of an auto-potentiating CNS based neurogenic pressor mechanism that becomes increasingly important in sustaining hypertension [13], [14]. Further, the chronic pressor effect of peripheral and central Ang II is amplified by raising salt intake [15], [16], and requires activation of brain mineralocorticoid receptors (MR) by local aldosterone and/or corticosterone [14].

Chronic actions of Ang II on BP have been linked also with endogenous sodium pump inhibitors. Although largely ignored, there are numerous documented interactions between brain aldosterone, Ang II and ouabain-like materials whose significance remained unclear [17]–[21]. Recently, it was proposed that the chronic pressor pathway for Ang II depends upon a neuromodulatory pathway involving aldosterone synthesis, MR, epithelial sodium channels, and increased activity of endogenous ouabain (EO) in the brain [14]. The primary outflow from this central pathway is increased SNA and the above-mentioned peptide hormones while other distal mechanisms that might contribute to Ang II hypertension have remained an enigma.

Here, we test the hypothesis that sustained elevation of central Ang II raises the circulating levels of endogenous ouabain-like compounds and reprograms key ion transporters that upregulate long-term arterial function. We also examine the roles of brain MR and cytochrome P-450 aldosterone synthase (B2) in these Ang II-evoked responses by using central (intracerebroventricular, icv) infusions of the MR blocker, eplerenone, and the selective B2 inhibitor, FAD286 ((+)-(5*R*)-4-(5,6,7,8-tetrahydroimidazo[1,5-*a*]pyridin-5-yl]benzonitrile hydrochloride) [22]. In each experimental paradigm, comprehensive quantitation and definitive analytical evidence regarding the response of the circulating compounds to central Ang II was obtained by radioimmunoassay (RIA), liquid chromatography (LC) and mass spectrometry (MS)-based methods.

## Methods

### Animals

Male Wistar rats (200 g) were obtained from Charles River Breeding Laboratories (Montreal, PQ, Canada). Housing was provided in a temperature-controlled environment with a 12∶12-hour light:dark cycle. Water and standard chow (0.3% NaCl) were provided *ad libitum*.

### Ethics

The present studies were carried out in full accordance with guidelines established by the Canadian Council on Animal Care and were approved by the University of Ottawa Animal Care Committee (HI-305) and also conformed to the guide for the Care and Use of Laboratory Animals published by the US National Institutes of Health (NIH publication 8^th^ Edition, 2011). All surgical procedures were carried out under isofluorane anesthesia and euthanasia was performed by decapitation.

### Formulations, Dosages and Central Infusions

All infused agents were dissolved in artificial cerebrospinal fluid (aCSF) sterilized with Acrodisc syringe filters (Pall, Life Sciences, Mississauga, Ontario CA). The composition of the aCSF was (in mmol/l) 121 NaCl, 3.4 KCl, 1.2 MgCl_2_, 0.6 NaH_2_PO_4_, 29 NaHCO_3_, and 3.4 glucose. The pH was 7.4, and osmolality was 296 mosm/kg. After a 1-wk acclimation, under isoflurane inhalation a stainless steel right-angle cannula was implanted into the left lateral cerebral ventricle and fixed to the skull of the rat with acrylic cement [23]. The upper (free) end of the cannula was connected to an osmotic minipump (Alzet model 2002, Durect Corporation, Cupertino, CA) for chronic ICV infusion at 0.5 µl/h for 14 days. Pumps were filled with aCSF (C; vehicle control), or with aCSF containing Ang II (A, 2.5 ng/min) alone or combined with eplerenone with 4% acetonitrile in aCSF (E, 5 µg/day; Pfizer, Canada) or FAD286 (F, 25 µg/day) (4 groups in total, n = 7–8/group) and implanted subcutaneously on the back of the rat. FAD286 is a single (+)-enantiomer (Novartis Institutes for BioMedical Research, Cambridge, MA). In the present study FAD286 was formulated as the hydrogen tartrate salt which is soluble in aCSF; the quantity infused is equivalent to the free base (1.67 mg FAD286 hydrogen tartrate = 1 mg of FAD286). The icv infusion rates for FAD286 and eplerenone were based on previous studies [20]. The icv-infused doses have no effect when infused peripherally [24].

### Blood Pressure

On day 12 of icv infusion, under isofluorane anesthesia, a polyethylene PE10/PE50 catheter was placed in the right femoral artery and was tunneled to the back of the neck. The following morning about 18 h after the surgery, the catheter was connected to a pressure transducer linked to a Grass polygraph (Model 7E) and a Grass 7P44 tachograph. Real-time digital data were obtained using a PC equipped with a Grass data acquisition and analysis program (Polyview 2.0). Rats were rested for 30 min, and then resting mean arterial pressure (MBP) and heart rate (HR) were recorded for 5 min. After the measurement, rats were returned to their original cages, where they stayed overnight in a quiet room until euthanasia and final tissue collection the following morning.

### Blood and Tissue Collection

On the morning of day 14, taking care not to disturb the animals, the rats were taken one by one to the necropsy room and decapitated. Trunk blood was collected directly into pre-chilled falcon tubes containing heparin. Plasma was obtained following centrifugation, and was stored at −70°C prior to solid phase extraction (SPE). The aorta from a euthanized rat was rapidly removed and placed in ice-cold physiological salt solution. The arteries were cleaned of fat and connective tissue, de-endothelialized, frozen in liquid nitrogen, and stored at −80°C before protein extraction, as described [25].

### Measurement of Endogenous Ouabain

All plasma samples were first subjected to SPE. Tissue samples were weighed, homogenized in methanol-water mixtures, vacuum dried, and extracted using C18 columns (200 mg, Bond Elut, Agilent Technologies). Subsequently, three assay methods were used in sequence as follows: 1) Routine SPE-RIA; reconstituted samples were employed in duplicate for ouabain RIA using the R7 antiserum described elsewhere [26], [27]. 2) SPE-LC-RIA; when significant differences were detected among the study groups by RIA, equivalent volumes of residual sample extracts from each animal were pooled according to their study group and separated by analytical HPLC using Ultracarb5 ODS 30, 250×4.6 mm C18 columns (Phenomenex, Torrance, CA). Vacuum dried fractions were reconstituted to be 10x their original plasma concentration and reassayed by ouabain RIA. 3) SPE-LC-MS3; residual 10x concentrated samples from the LC-RIA step were mixed vol:vol with an 80∶20 CH_3_CN:water solution containing 250 µM LiCO_3_ and the internal reference steroid dihydroouabain (DHO, 25 nM final) and analyzed by offline multistage mass spectrometry (i.e., SPE-offline LC-MS-MS-MS). Key MS3 product ions for EO and DHO were monitored at 379.2 and 381.2 m+Li^+^/z, respectively using a Bruker HCT Ultra ion trap. EO was quantitated relative to the intensity of the DHO reference ion. Additional details for the various methods have been described [28].

### Immunoblotting for Arterial Proteins

Membrane proteins were solubilized in sodium dodecyl sulfate buffer containing 5% 2-mercaptoethanol and separated by polyacrylamide gel electrophoresis as previously described [25]. The following antibodies were used: rabbit polyclonal anti-TRPC6 (dilution 1∶500, Sigma-Aldrich, St Louis, MO); mouse monoclonal anti-NCX1 (dilution 1∶500; R3F1; Swant, Bellinzona, Switzerland); mouse monoclonal anti-SERCA2 (sarco−/endoplasmic reticulum Ca^2+^ pump; dilution 1∶1,500; Affinity Bioreagents, Golden, CO). Gel loading was controlled with monoclonal anti-β-actin antibodies (dilution 1∶10,000; Sigma-Aldrich). After washing, membranes were incubated with anti-rabbit horseradish peroxidase-conjugated IgG for 1 h at room temperature. The immune complexes on the membranes were detected by enhanced chemiluminescence plus (Amersham Biosciences, Piscataway, NJ) and exposure to X-ray film (Eastman Kodak, Rochester, NY). Quantitative analysis of immunoblots was performed by using a Kodak DC120 digital camera and 1D Image Analysis Software (Eastman Kodak). In these studies we used aorta; the blotting of low abundance membrane proteins in arterioles <300 µm is often not feasible without pooling vessels from large numbers of animals. In general where smaller arteries have been studied simultaneously with aorta the primary changes in NCX1 are similar [28], [29].

### Statistical Analyses

The numerical data are means ± SEM with the number of animals as indicated. Immunoblots were repeated at least four to six times for each protein. Statistical significance was determined using one way ANOVA followed by *t*-test with the Bonferroni correction, or non-parametric analyses as appropriate. *P*<0.05 was considered significant.

## Results

### Central infusion of Angiotensin II raises plasma endogenous ouabain and BP

**Figure 1:**
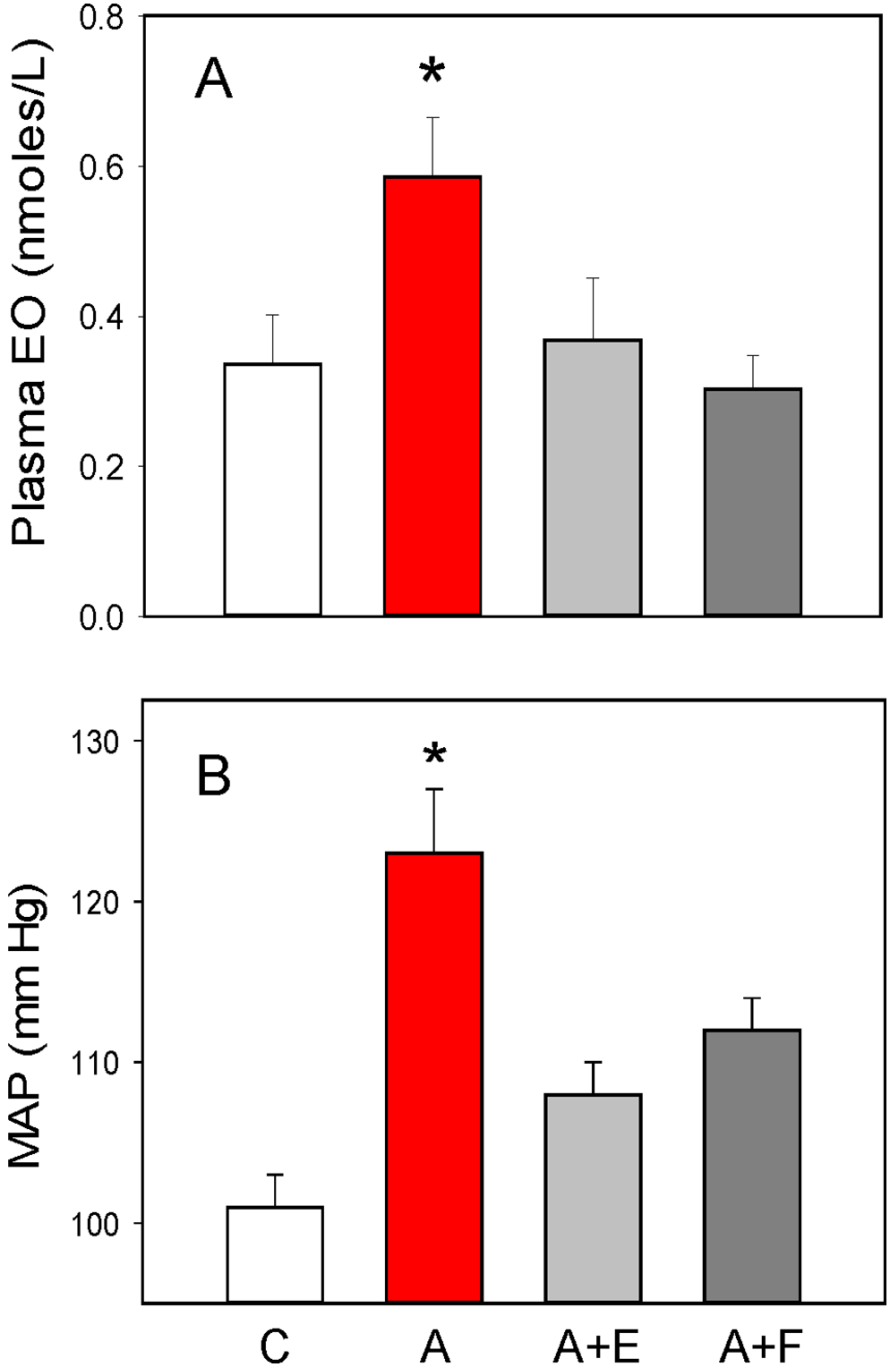
Plasma EO and BP are elevated following a 14 day icv Ang II infusion: role of central mineralocorticoid receptors and aldosterone synthase. Panel **A**, plasma EO. Panel **B**, mean arterial blood pressure. C = vehicle control; A = Ang II; A+E = Ang II + eplerenone; A+F = Ang II + FAD-286; *P<0.01 vs all other groups; n = 7–8/group.

**Figure 1** shows that prolonged icv infusion of Ang II significantly increased the concentration of plasma EO (**Panel A**), as measured by routine SPE-RIA, and significantly raised mean arterial BP (**Panel B**). Central-co infusions of either eplerenone or FAD286 blocked the stimulatory effect of icv Ang II on plasma EO and reduced the rise in BP by 60–70%. The pattern of BP changes is similar to those recently reported using telemetry [30].

### Central Angiotensin II increases the arterial myocyte expression of Ca^2+^ transport proteins

**Figure 2:**
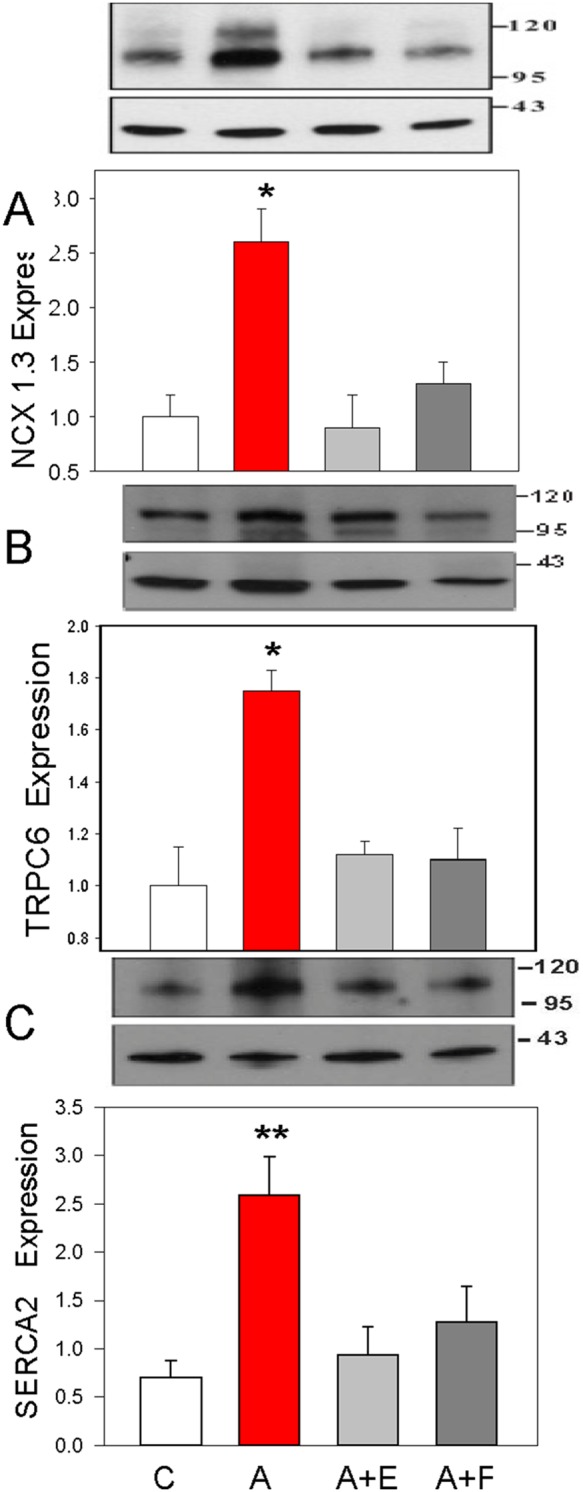
Increased expression of arterial Ca^2+^ transport proteins following a 14 day icv Ang II infusion. **Panels A**, **B and C**, representative Western blots and summary data for NCX1, TRPC6 and SERCA2 expression, respectively, in de-endothelialized aorta. Blots were normalized for β-actin as a loading control and the changes shown are relative to the control group. All lanes were loaded with 10–20 µg of membrane protein. The expected molecular weights are: NCX1, 116 KDa; TRPC6, 111 KDa, SERCA2 115 KDA, and β-actin, 42 KDa. C = vehicle control; A = Ang II; A+E = Ang II + eplerenone; A+F = Ang II + FAD-286; *P<0.01 vs all other groups; n = 7–8/group. Summary data are from 4 blots for each protein.

**Figure 2** shows the expression of **A**: NCX1, **B**: TRPC6 and **C**: SERCA2 proteins in the aortic myocytes of the four study groups; icv infusion of Ang II increased expression of the three proteins by 2.6, 1.75 and 3.7-fold, respectively. These effects of Ang II were blocked by central co-infusion with either eplerenone or FAD286.

### Effect of central Ang II on plasma EO as determined by LC-RIA

**Figure 3:**
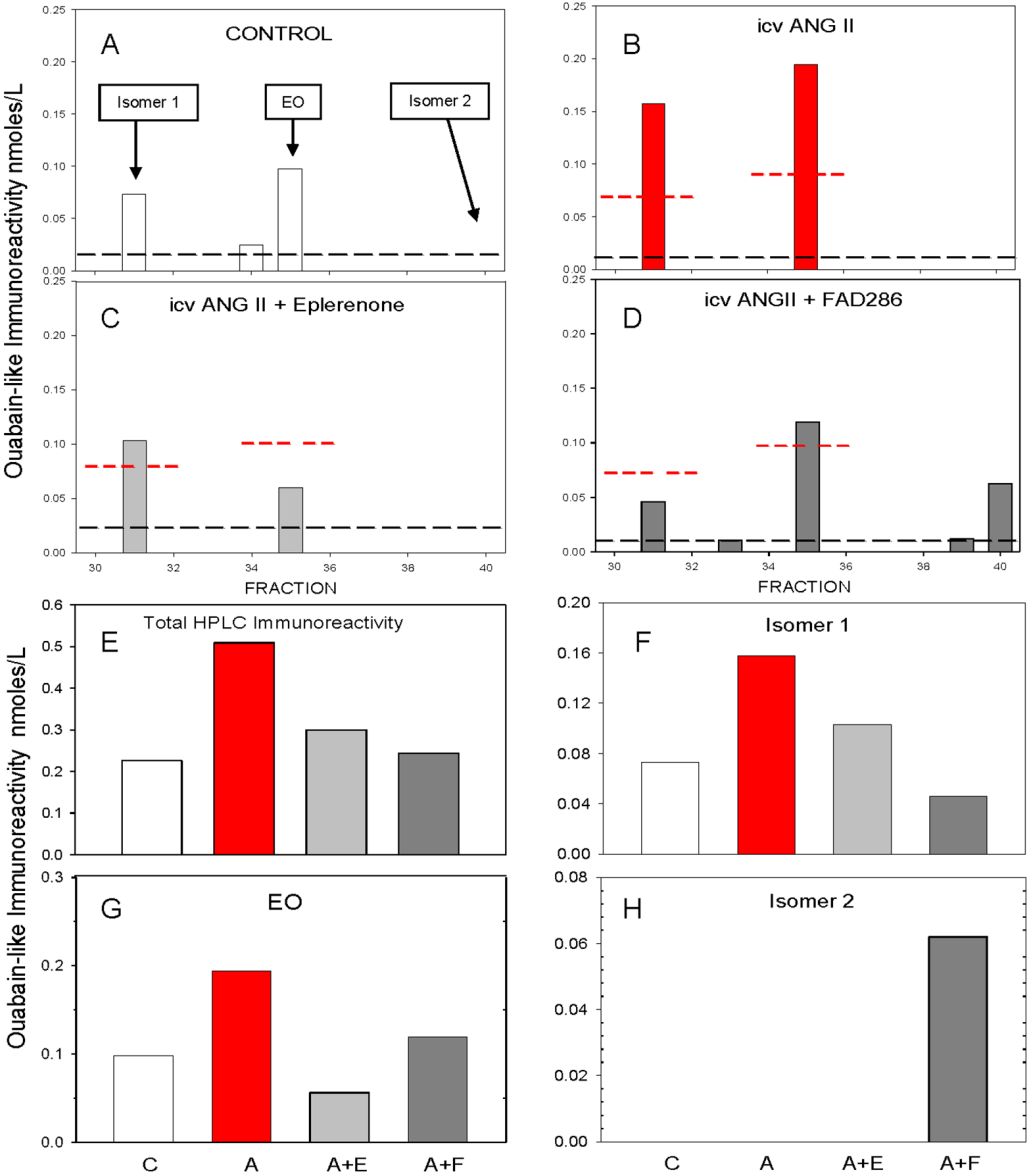
Summary analysis of the effects of CNS Ang II infusion on plasma EO based upon SPE-LC-RIA data. Ouabain immunoreactivity in LC separated plasma pools from controls (**A**), icv Ang II (**B**), icv ANG II + eplerenone (**C**), and icv Ang II + FAD286 infused rats (**D**). In each case EO eluted in fraction 35 (black bar). Other immunoreactive materials are shown in the gray bars. Dashed red lines in **A–D** are the values from the control for comparison. Dashed lines represent 5% threshold for the RIA under the conditions used. Only peaks that exceeded the thresholds are shown. Ouabain immunoreactivity in LC separated plasma pools arranged by study group. Response of: total ouabain immunoreactivity (**E**), Isomer 1 (**F**), EO (**G**), and Isomer 2 (**H**). Study groups: C, icv vehicle. A, icv Ang II. A+E, icv Ang II + eplerenone. A+F, icv Ang II + FAD 286.

The initial determination of the response of plasma EO to icv Ang II, was made by SPE-RIA (**Fig. 1**). To verify that the immunoreactive substances detected by the RIA were related to EO, we separated pooled SPE samples from each icv treatment group by HPLC. The resultant fractions were screened by RIA as shown in **Fig. 3**. The panels show the ouabain-like immunoreactivity in each of the HPLC fractions from the four study groups; i.e., **A**: control, **B**: Ang II, **C**: Ang II + eplerenone, and **D**: Ang II + FAD286. Ouabain immunoreactivity is shown only when it exceeds twice the threshold sensitivity of the RIA (dashed lines) in the relevant conditions. In each case, the immunoreactivity in fraction 35 is EO and this corresponds to the same elution time as commercial ouabain (not shown). Additional peaks of immunoreactivity were routinely detected in fractions 31 (isomer 1) in all groups, and in fraction 40 (isomer 2) in the A+F group; isomer 2 was below the detection threshold for the RIA in the other three groups. As proven by subsequent mass spectral analyses (**File S1** Results and Figures S1 and S2), the materials in fractions 31 and 40 are isomers of EO and therefore are labeled accordingly.

**Figure 3** **panel** **E** presents a summary of the total immunoreactivity recovered from all the LC fractions in each of the four study groups. For each group, the summed immunoreactivity was in reasonable agreement with the total immunoreactivity measured by the routine SPE-RIA (**Fig. 1A**). Specifically, in each pool, 70–80% of the injected immunoreactivity was accounted for.

**Panels** **F**, **G** and **H** in **Fig. 3** present, respectively, the summed immunoreactivities for isomer 1, EO, and isomer 2 according to the icv treatment groups. In response to icv Ang II, the levels of isomer 1 and EO increased and the effect was blocked by eplerenone and FAD286. Isomer 2 was observed only in the A+F group. Thus, the effect of A and of eplerenone could not be determined from the RIA data. As these results were obtained from pools made by combination of very small amounts of sample, statistical analyses are precluded. Nevertheless, the data from the summary SPE-LC-RIA analysis (**Fig. 3** **,** **Panel E**) compares favorably with the total ouabain immunoreactivity (**Fig. 1A**) and confirms that icv Ang II raises the circulating levels of EO and isomer 1. The LC-RIA results also confirm that eplerenone and FAD286 block the increase in EO evoked by icv Ang II.

### Effects of central Ang II on plasma EO as determined by LC-MS3

**Figure 4:**
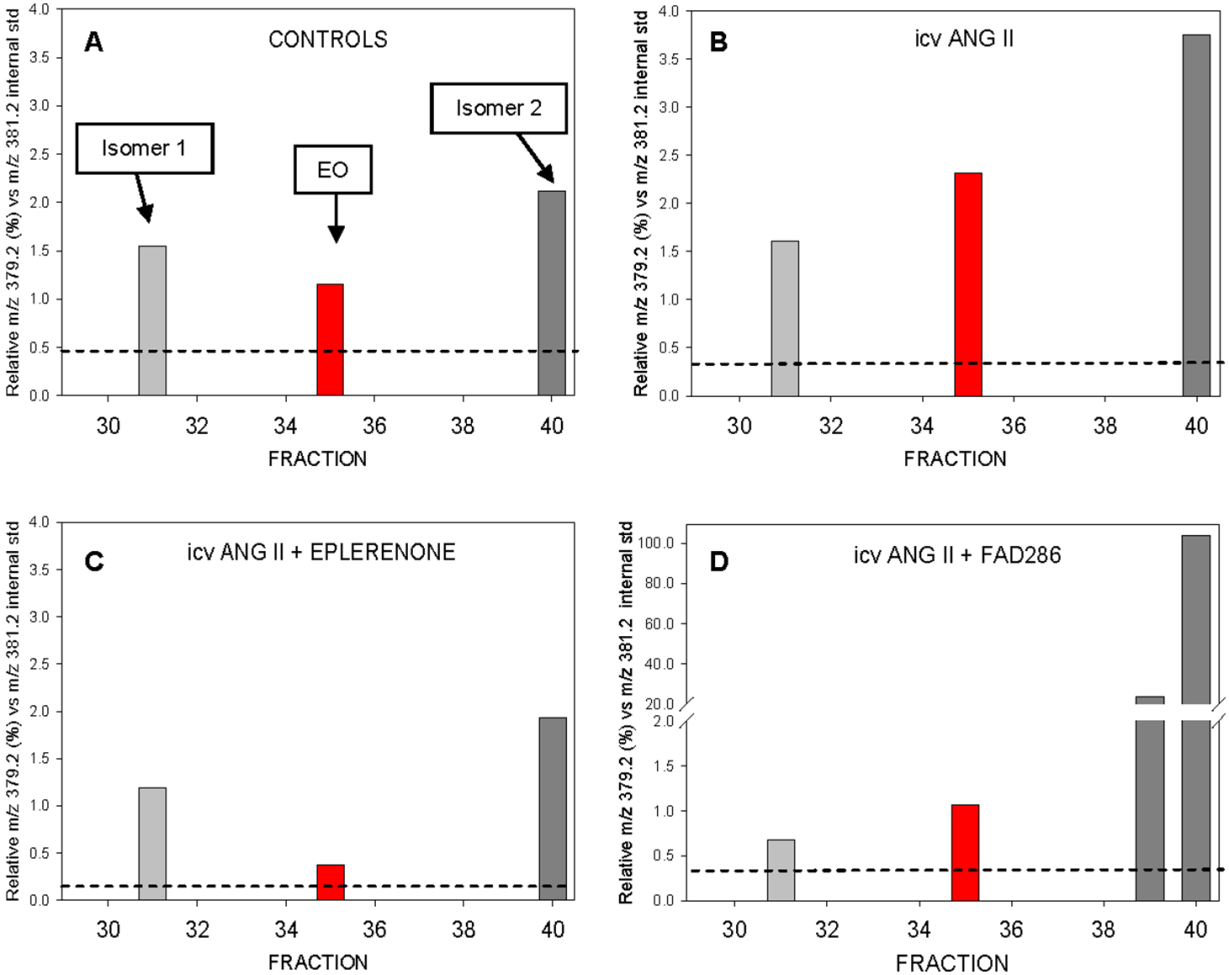
Summary analysis of plasma EO and isomers in response to CNS Ang II infusion based upon SPE-LC-MS3 data. In each case, the relative spectral intensities at m/z 379.2 are shown normalized to the DHO reference at m/z 381.2 for each of the LC fractions. The study groups were: **A**; Controls. **B**; icv Ang II infused. **C**; icv Ang II + eplerenone. **D**; icv Ang II + FAD286. The dashed lines in each plot represent the thresholds for detection (i.e., >3x ambient signal to noise ratio).

The observation of multiple peaks of ouabain immunoreactivity prompted interrogation of the LC fractions by multistage mass spectrometry (MS3). **Figure S1** (and **File S1** Results) shows MS3 molecular product ion scans for HPLC fractions derived from the icv Ang II infused group that either lack (e.g., **F30**) or contain ouabain immunoreactivity (**F31, F35, F40**). In the case of the plasma extracts, MS3 product ions at 379.2 m/z were observed only in those fractions (**F31, F35** and **F40**) that also exhibited the most prominent ouabain immunoreactivity (c.f., **Fig. 3**); this molecular ion was not detected in adjacent fractions that lacked immunoreactivity (e.g., **F30**). **Figure S2** (and File S1) shows the relevant portions of the MS3 spectra from **Fig. S1** that have been enlarged for clarity. **Figure 4** presents the summary data from the MS3 analyses of the HPLC fractions according to the study group (**Fig. 4** **A–D**). EO and isomers 1 and 2 were detected in all groups and the relative amounts of these steroids in the control and icv Ang II groups, respectively are shown in **Panels A** and **B**. In response to icv Ang II (**B**), levels of EO and isomers 1 and 2 increased; this effect was blocked when eplerenone (**C**) was co-administered with Ang II. Addition of icv FAD286 to the Ang II infusate blocked the increase in EO and isomer 1 (panel **D**). In the case of isomer 2, the addition of FAD286 had a dramatic effect; levels of isomer 2 increased >50 fold above controls and the ion intensities for this isomer (**Fig. S2 E**) exceeded the DHO reference ions. The calculated plasma concentrations of isomer 2 in the icv groups were; controls, 0.3; icv Ang II, 0.55; icv Ang II+E, 0.3; and Ang II+F, 18 nmoles/L.

Together, the MS results in **Fig. S2 and** **Fig. 4** show that the prominent immunoreactivities detected by the RIA in **Fig. 3** are EO and its isomers. They demonstrate also that isomer 2 cross-reacts to a minimal extent (<10%) with the anti-ouabain antibodies in the RIA compared with either EO or isomer 1. Thus, the total immunoreactivity ordinarily detected by our routine RIA for EO (e.g., **Fig. 1A**) consists primarily of EO and isomer 1. The MS3 data prove also that the ouabain immunoreactive materials in fractions 31, 35 and 40 are structurally related. For example, the immunoreactive materials in fractions 31 and 40 have identical m/z values for the key primary and product ions; i.e., they are isomers of EO. The MS3 spectra for isomer 2 included an additional unique, prominent molecular ion at 417.2 m/z (**Fig. S2 E**). This highly abundant ion was specific for isomer 2 and was not found in the native spectra for either EO or isomer 1 (not shown). Note, however, that a weak MS3 background product ion at 417.2 m/z of uniform low abundance was observed in the spectra for all LC fractions. This low level background ion arises from the DHO internal reference.

Adrenal and pituitary levels of EO were also measured by routine SPE-RIA (**Table 1**). Relative to the vehicle controls, all three animal groups infused with icv Ang II tended to have a diminished adrenal content of EO while their pituitary EO contents trended upward. As the group differences were not statistically significant, and the residual amounts of the SPE samples were small, no further analytical work was performed.

**Table 1:** Adrenal and Pituitary Levels of EO.

Group	n	Adrenal EO (µg/Kg wet wt)	Pituitary EO (µg/Kg wet wt)
Control	7	0.59±0.11	2.31±0.39
icv ANG II	8	0.43±0.11	2.74±0.77
icv ANG II + Eplerenone	7	0.46±0.08	3.23±0.52
icv ANG II + FAD286	8	0.37±0.13	3.76±0.66

Means ± sem from routine SPE-RIA measurement.

## Discussion

The long term pressor effect of central Ang II is associated with the progressive activation of a slow neuromodulatory pathway [14]. This pathway is mediated by a sequence that involves local aldosterone synthesis, activation of MR, and benzamil-sensitive epithelial sodium channels (ENaCs), increased ENaC activity and local synthesis of brain ouabain-like compounds [14], [20], [30], [31].

**Figure 5:**
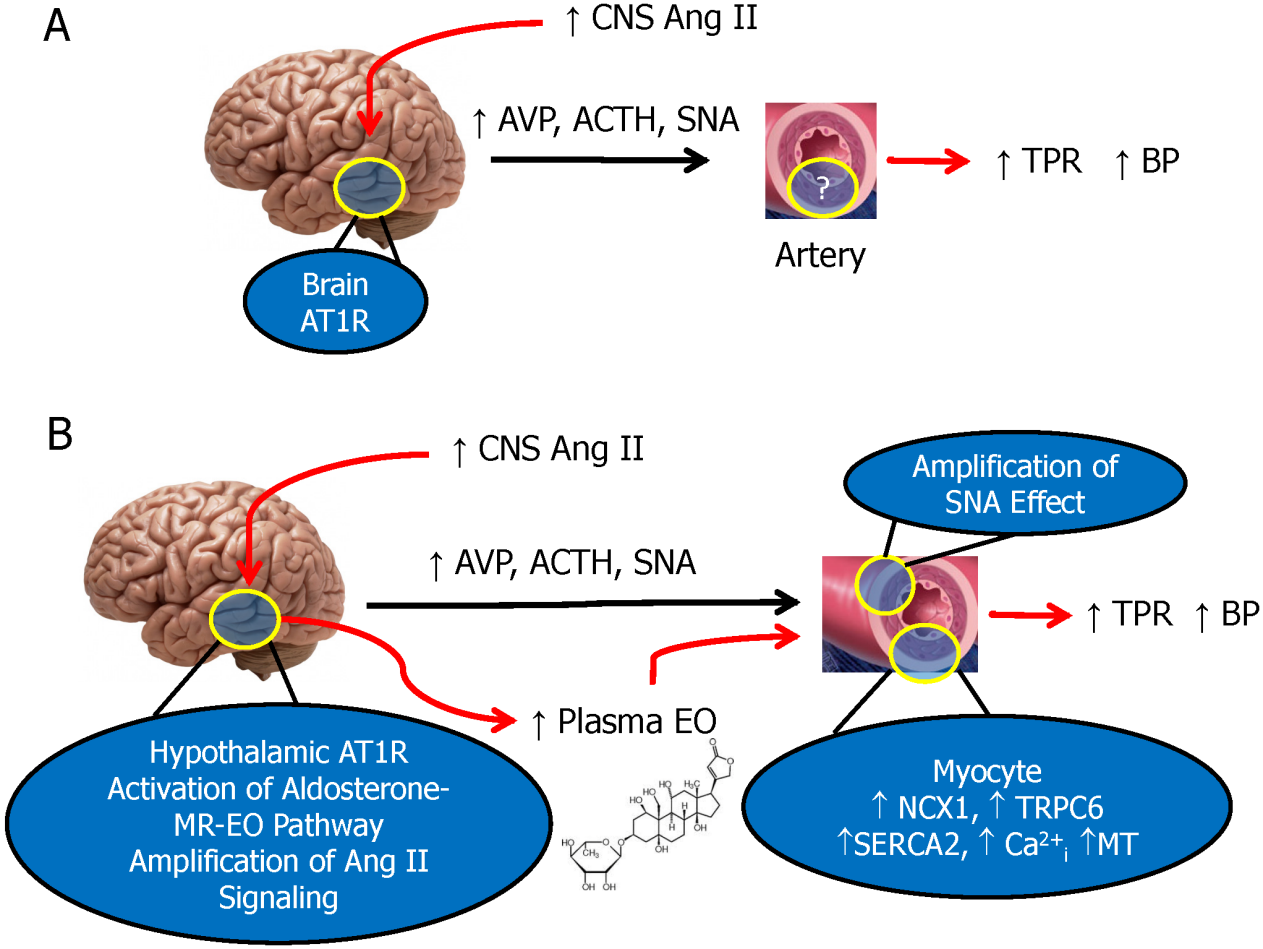
Proposed scheme for the chronic pressor action of central Ang II in the rat. **A.** Summary of prior ideas in which the links between icv Ang II and elevated BP were mediated by activation of angiotensin type I receptors (AT1R), increases in sympathetic nervous system activity (SNA), and elevated plasma levels of AVP and ACTH [2]–[5]. **B.** Revised model presenting a pathway involving the CNS, humoral and vascular events that may link activation of hypothalamic AT1R with hypertension as suggested by the present and related studies. The CNS component involves amplification of chronic Ang II mediated signaling via local stimulation of mineralocorticoid receptors (MR) by aldosterone and increased brain and circulating EO [14]. The CNS effects of Ang II raise SNA and plasma EO; the latter reprograms arterial myocyte function leading to: a) Increased Ca^2+^ and Na^+^ entry via NCX1 and TRPC6, and increased intracellular Ca^2+^ stores via SERCA2 [29], [54]–[56], [59]. b) Amplification of SNA in the sympathetic ganglia and vascular wall [60], [61] and, c) increased myogenic and evoked tone, and elevated TPR and BP [43], [71]. A central MR antagonist blocks all measured downstream effects of icv Ang II. The increased SNA and vascular reprogramming together appear to underlie the elevated BP. AVP, plasma arginine vasopressin. ACTH, adrenocorticotropic hormone. MT, myogenic tone. TPR, total peripheral resistance. See text for references and abbreviations not listed here.

The present study focused exclusively on the effects of prolonged central Ang II administration in the rat using a single time point when the slow neuromodulatory pathway is known to be maximally active. The study generated four major new findings with regard to key CNS and peripheral components involved in the response. First, sustained central infusion of Ang II elevates circulating EO (**Fig. 1A**), a vasopressor steroid, and augments the expression of three key proteins involved in Ca^2+^ and Na^+^ transport, and in Ca^2+^ homeostasis and signaling, in arterial myocytes (**Fig. 2**). Second, all the humoral, vascular and hemodynamic effects of icv Ang II we observed were prevented by the central administration of eplerenone, a potent and highly selective MR blocker [32], and, to a lesser extent, by inhibition of aldosterone synthase by FAD286 [22]. This demonstrates that all the measured peripheral variables are subject to regulation by the CNS. Third, in addition to EO, LC-RIA results showed that icv Ang II raised the plasma levels of a chromatographically-distinct ouabain-immunocrossreactive material. Analysis by mass spectrometry showed that this material was a polar isomer of EO (‘isomer 1’). A second, less polar isomer (‘isomer 2’) was also observed by LC-MS3, but was minimally immunocrossreactive and not routinely detected by our ouabain RIA. Mass spectrometry confirmed that icv Ang II increased circulating EO, and also that icv eplerenone and FAD286 blocked this effect. MS analyses showed also that the plasma levels of isomer 2 were elevated dramatically (>50-fold) in rats given icv Ang II together with the aldosterone synthase inhibitor FAD286. Fourth, when taken together, our results demonstrate that the sustained elevation of brain Ang II activates a novel long-range neurohumoral-vascular control axis. This axis (**Fig. 5**) forms part of the long term pressor pathway for brain Ang II and employs CNS aldosterone, MR signaling and other peripheral mechanisms that amplify the central effects of Ang II [14]. The peripheral elements of this axis include elevated circulating levels of EO and up-regulated expression of three key ion transport proteins in arterial myocytes. We suggest these hitherto undescribed events contribute to the ability of chronic central Ang II to maintain elevated BP.

### Central Control of Sodium Pump Inhibitors

Endogenous Na^+^ pump inhibitors have been linked with the brain and its influence on BP. Early work suggested that CNS Ang II influenced the peripheral levels of ouabain-like substances [18], [33], [34]. Moreover, Na^+^ pump inhibitory activity was missing from the circulation of volume-expanded rodents with electrolytic lesions of the anteroventral third ventricular (AV3V) area of the hypothalamus [35]; also, the lesioned animals failed to develop hypertension [36]. Furthermore, the central administration of 6-hydroxydopamine lowered hypothalamic and plasma ouabain-like activity, whereas peripheral sympathectomy had no effect [33], [34]. This suggested an important role for central, but not peripheral, catecholaminergic and/or dopaminergic neurons. However, the significance of the aforementioned observations remained vague; early studies often were compromised by highly non-specific assays, and the chemical nature of any humoral inhibitor was unknown. Following identification of endogenous ouabain [37], subsequent work showed that the chronic elevation of plasma and CNS ouabain raised blood pressure [19], [38]–[41]. Specific immunoassay methods were introduced [26] and refined [27], culminating in two- [42] and, most recently, three-dimensional mass spectrometry [28]. Recent insights into vascular ion transport pathways in salt-sensitive hypertension [43], and the elucidation of new central components that regulate the long-term control of BP by the brain [14], prompted us to reexamine possible interrelationships between the CNS, sodium pump inhibitors, and vascular ion transport pathways in rats infused with icv Ang II using highly advanced analytical methods.

The present work shows that the CNS Ang II–Aldosterone–MR modulatory pathway up-regulates circulating EO and thereby stimulates downstream arterial myocyte mechanisms already known to raise vascular tone and long-term BP [43]. Brain Ang II plays important roles in thirst, fluid and electrolyte homeostasis and long term BP regulation [44]. In addition, aldosterone is synthesized in the brain [45]–[48] and is stimulated by Ang II. Aldosterone activates local MRs, which stimulate benzamil-sensitive epithelial sodium channels and central production of EO. Similarly, brain ouabain has important effects on cardiovascular function [27], [41]. Thus, central pharmacological blockade of any of the above noted factors (aldosterone synthase, MRs, or conjugation of brain ouabain with high-affinity antibodies) prevents sympathetic hyperactivity and blocks salt-sensitive and Ang II-induced hypertension [14], [20], [23], [30].

### Elevated EO, Arterial Ca^2+^ Signaling and Hypertension

In rodents, the primary sustained elevation of plasma ouabain into the low nanomolar range raises BP [27], [38], [39], [41]. In humans, elevated levels of EO circulate in ∼45% of patients with essential hypertension (EH), and in a large fraction of patients with primary hyperaldosteronism due to adenoma but not hyperplasia [27], [49]. In contrast to EH, the spontaneously hypertensive rat has low circulating EO, suppressed arterial NCX1 activity, and as expected, no BP reduction in response to immunoneutralization of EO [50]–[52]. The aforementioned experimental and clinical studies demonstrate that EO can induce hypertension and that elevated EO is not a consequence of hypertension. Further, studies in normal and genetically-modified mice, and cultured arterial myocytes, have elucidated the mechanism by which EO as well as *ex* *vivo* ouabain raises vascular tone. This mechanism involves: *i.* binding of ouabain/EO to arterial myocyte Na^+^ pumps with an α2 catalytic subunit [53]; *ii.* augmented Ca^2+^ and Na^+^ influx mediated via chronic up-regulated expression of vascular myocyte Na^+^/Ca^2+^ exchanger-1 (NCX1) and receptor-operated channels (ROCs, especially those with a TRPC6 subunit); *iii.* increased cytoplasmic Ca^2+^; and *iv.* elevated myogenic tone and vascular reactivity [29], [43]. This same ouabain-activated Ca^2+^ signaling pathway operates in human arterial myocytes [54], and is activated in Milan hypertensive rats, a model with high circulating EO [55],[56]. The Ca^2+^ signaling pathway has key roles in ouabain-induced, ACTH-evoked, and some salt-sensitive forms of hypertension [53], [57]–[59]. Notably, animals in which arterial NCX1 is knocked out fail to develop Ang II hypertension while their overexpressing counterparts develop higher than normal BP [59]. Thus, up-regulation of arterial NCX1 and Ca^2+^ signaling *in* *vivo* is a critical pressor mechanism in experimental models of hypertension where EO is elevated [55]–[58]. The same Ca^2+^ signaling events are up-regulated by nanomolar ouabain in primary cultures of human and rodent arterial myocytes [29], [54]. This *ex* *vivo* demonstration shows also that the changes in **Figures 1** and **2** are likely direct effects of the elevated EO *per se*; they are unlikely to be a consequence of hypertension or secondary to other humoral or neural factors *in* *vivo*.

In addition to the central effects of Ang II on SNA in the rat, increases in circulating EO act peripherally to further amplify sympathetic effects by at least two mechanisms. In the superior cervical ganglion, *in* *vivo* ouabain amplifies synaptic transmission [60] and sympathetic traffic reaching the artery wall is further amplified locally by nanomolar ouabain [61]. Thus, central Ang II activates MR-dependent central mechanisms [14] that stimulate SNA and, as shown here, raise circulating EO. In turn, the elevated EO upregulates multiple arterial myocyte proteins involved in Ca^2+^ signaling that further augment the effects of the increased SNA. We suggest that these previously unrecognized humoral and arterial mechanisms contribute to the sustained pressor effects of central Ang II (**Fig. 5**).

Ouabain also reduces nitric oxide signaling in the kidney [62], and shifts the pressure-natriuresis relationship rightward [40]. Thus, the combination of the vasopressor and renal effects of elevated circulating EO is expected to maintain a large portion of the elevated BP in Ang II hypertension. Further studies will be required to determine the quantitative contribution of EO to the acute and chronic phases of central Ang II hypertension.

### Quantitative and Qualitative Analyses of EO and Isomers

In addition to routine RIA, the present work employed HPLC-RIA and offline HPLC-MS3 to provide unambiguous and specific evidence that plasma EO is elevated by brain Ang II. The latter are summarized in the supporting information (**Figs S1, S2** and **File S1** Discussion). In addition, a number of deductions can be made about the general structures of the isomers from the MS spectra (**File S1** Structure-activity Relationships for EO isomers).

### Central vs Peripheral Biosynthesis of EO and its isomers

CNS synthesis of ouabain-like materials has been suggested as a source of local CNS EO and also as a possible source of circulating EO [34], [63]. The biosynthetic pathway for EO and aldosterone in the adrenal are similar [64]; both steroids are also likely synthesized in the CNS at the same sites with the same hydroxylases. The enzymes for corticosterone and aldosterone synthesis [45], [65], cytochrome P-450 11β-hydroxylase (B1) and cytochrome P-450 aldosterone synthase (B2), respectively catalyze 11, 18 and 19 hydroxylation and (B2) 18-oxidation to produce aldosterone [66]. Although CNS synthesis of EO and its isomers is likely, the expression of B2 (and B1) is considerably (10–500 times) lower in the CNS versus the adrenal [45], [47], [66], [67]. Further, under normal circumstances, the majority of circulating EO appears to be of adrenocortical origin [27]. Similarly, the extraordinarily large amounts of isomer 2 in the circulation of the FAD286-infused animals (**Figs. S2** and **4**) may imply that a central source for this new steroid is unlikely. Nevertheless, icv 6-OHDA suppresses circulating ouabain-like activity [33], [34], indicating a dominant effect of central catecholaminergic and/or dopaminergic neurons. In addition, unlike aldosterone and corticosterone, which are removed rapidly from the circulation and are replaced by correspondingly high rates of adrenal biosynthesis, EO (ouabain) is cleared relatively slowly in the rat [38]. Thus, CNS expression of B2 may be sufficient to explain the increase in circulating EO evoked by icv Ang II.

In response to icv FAD286, there was a dramatic increase in the circulating level of isomer 2. Thus, in addition to blocking aldosterone biosynthesis, FAD286 has other actions. The R enantiomer of FAD286, a selective B2 inhibitor, has no major effect on corticosterone production, but blocks the ability of Ang II to raise plasma aldosterone [30], [68] and EO (**Fig. 1**). The latter effect of FAD286 likely reflects inhibition of 11 and/or 19 hydroxylation in EO and, in turn, raises the possibility of a pathway in which 11-deoxycorticosterone (DOC) normally rate limits EO formation. Hence, a straightforward explanation for the effect of FAD286 is that central inhibition of B2 leads to: a) loss of the Ang II-stimulated CNS EO synthesis and no increase in plasma EO and, b) an increase in brain DOC that fuels isomer 2 synthesis. Thus, our data clearly imply that both EO and isomer 2 are made in the CNS.

The elevation of plasma corticosterone and aldosterone by icv Ang II [30] reveals a signaling pathway from the brain to the adrenal cortex. This pathway is most likely mediated by ACTH [9]. Further, ACTH stimulates the secretions of aldosterone, cortisol and EO from adrenocortical cells and this might explain the rise in circulating EO [69]. However, the observation that icv FAD286 does not suppress plasma aldosterone [24], indicates that leakage of central FAD286 to the adrenal is minimal. Thus, the effect of icv Ang II on plasma EO and the impact of FAD286 on isomer 2 production (**Fig. 4D**) are best explained by increased biosynthesis of these steroids in the CNS and not the adrenal.

### Adrenal and Pituitary levels of EO

The adrenal EO content in animals infused with icv Ang II trended downward, while pituitary EO trended upward (**Table 1**). The simplest explanation is that the trends reflect the decreased demand for adrenal biosynthesis, with increased hypothalamic synthesis and pituitary secretion of EO, respectively. It is of interest that the trend was most prominent in the group infused with Ang II and FAD286; i.e., conditions where the plasma levels of isomer 2 were greatest.

The increase in the apparent pituitary level of “EO” in the FAD286 group likely reflects a contribution from isomer 2. Because the R7 ouabain antiserum has <2% crossreactivity with isomer 2, the actual molar contribution of isomer 2 to the measured EO in the FAD286 group may be very significant; i.e., 1–2 orders of magnitude larger (10–100 µg/Kg wet wt) than indicated by the increase in pituitary “EO”. Limited amounts of sample precluded further investigation of this issue by LC-MS3.

### Significance of EO and its Isomers

In these studies, variation in the plasma levels of isomer 1 and, especially, EO were best correlated with the BP response to icv Ang II (**Fig. 3**). The simultaneous elevation of plasma EO and aldosterone [30] is noteworthy; it was previously described in many patients with primary aldosteronism [49], and because these steroids may co-amplify their vasopressor effects [70]. Further, many clinical and experimental studies that demonstrated significant relationships with EO and hemodynamics and outcomes [27] used the R7 ouabain antiserum for EO RIA. As shown in **Figure 3A**, the bulk of the RIA signal is the sum of EO and isomer 1, both present in near equimolar amounts (i.e., ∼100 pM each). The basal levels of isomer 2 were quantitatively similar to EO and isomer 1 (**Fig. 4**), and can be observed (see **File S1** Quantitative and Qualitative Analyses of EO and Isomers) only with LC-MS [28]. Currently, lack of specific antisera for the isomers means that LC separation is desirable prior to RIA for independent quantitation of EO and/or isomer 1; this may not be practical in clinical studies were large numbers of patients are involved [42], [49].

In the present study, the increased plasma EO was associated with significant increases in arterial myocyte NCX1 and TRPC6 expression and BP, while the >50-fold increase in isomer 2 in the FAD286 group produced no such effects. Thus, any hypertensinogenic activity of isomer 2 must be<1/50^th^ that of EO. It is also possible that the high levels of isomer 2 in the FAD286 group might act as a receptor antagonist of EO and lower BP by that mechanism. In this study, however, the depressor effect of FAD286 is best correlated with its ability to normalize circulating EO (**Fig. 1**).

In summary, a novel neuroendocrine axis is associated with the sustained pressor effect of brain Ang II. The proximal components of this axis are neuronal pathways activated by brain Ang II that depend upon central aldosterone and MRs. The distal components of the axis include up-regulated circulating levels of EO and related steroids, and functional reprogramming of arterial function due to increased expression of arterial myocyte proteins that raise arterial myocyte Ca^2+^ and myogenic tone [71] and that augment sympathetic responses. Taken together, our results are consistent with the idea that long term increases in central Ang II and circulating EO sustain BP via the combined effects of heightened sympathetic activity and the functional reprogramming of arterial function.

## Supporting Information

Figure S1 **Identification and quantitation of plasma EO and isomers by SPE offline LC-mass spectrometry (MS).**
(TIF)

Figure S2 **SPE offline LC-mass spectrometry (MS) fractions showing MS3 Spectra for EO and two isomers and the effects of CNS Ang II infusion.**
(TIF)

File S1 **Supplementary Text Information for Results, Discussion and Figures.**
(DOCX)
