# Supplementary figures and images for: Neuroendocrine Humoral and Vascular Components in the Pressor Pathway for Brain Angiotensin II: A New Axis in Long Term Blood Pressure Control
Source: PLoS One. 2014 Oct 2;9(10):e108916. doi: 10.1371/journal.pone.0108916 (PMC4183521; doi:10.1371/journal.pone.0108916)

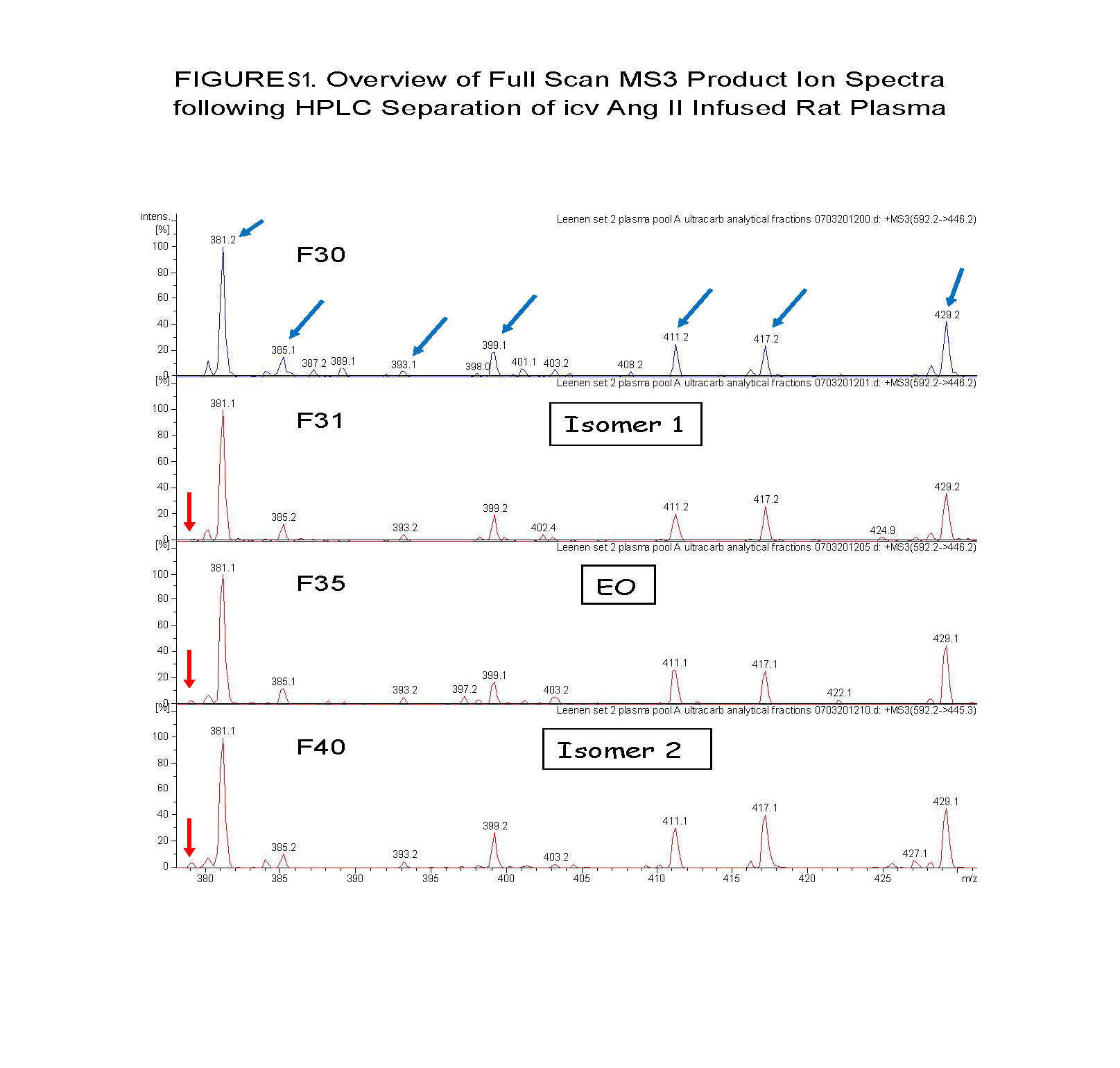

Supplement: Figure S1 — Identification and quantitation of plasma EO and isomers by SPE offline LC-mass spectrometry (MS). (TIF) [file pone.0108916.s001.tif]

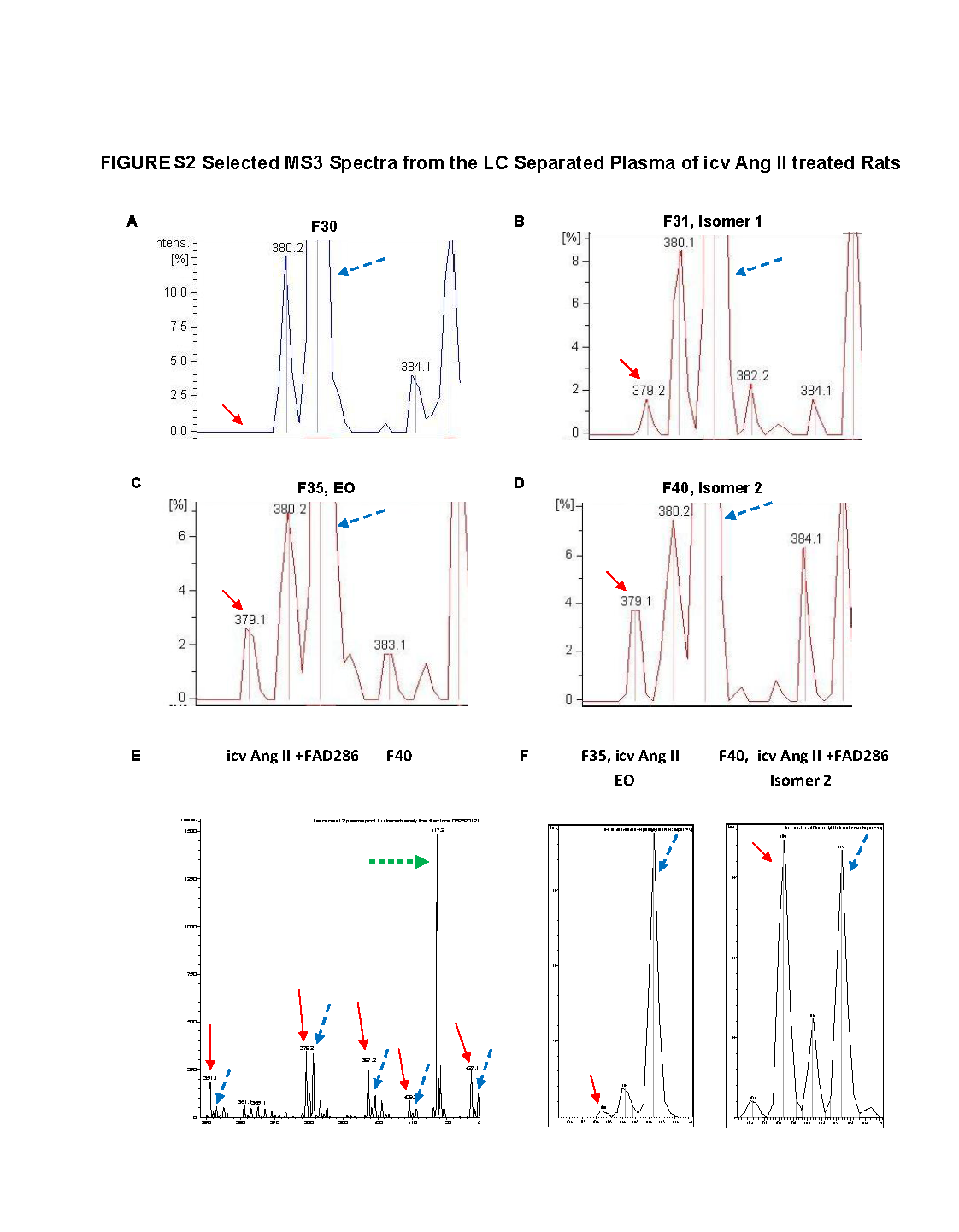

Supplement: Figure S2 — SPE offline LC-mass spectrometry (MS) fractions showing MS3 Spectra for EO and two isomers and the effects of CNS Ang II infusion. (TIF) [file pone.0108916.s002.tif]
