# Supplementary material for: Neuroendocrine Humoral and Vascular Components in the Pressor Pathway for Brain Angiotensin II: A New Axis in Long Term Blood Pressure Control
Source: PLoS One. 2014 Oct 2;9(10):e108916. doi: 10.1371/journal.pone.0108916 (PMC4183521; doi:10.1371/journal.pone.0108916)
Supplement: File S1 — Supplementary Text Information for Results, Discussion and Figures. (DOCX) [file pone.0108916.s003.docx]

**Supplementary Text Information for Results, Discussion and Figures.**

**Results**

In **Fig S1**, the blue arrows indicate the more prominent MS3 product ions (± 0.1 mass units) derived from the DHO internal reference (i.e., m/z 429.2, 417.2, 411.2, 399.2, 393.2, 385.2 and 381.2). EO and its two isomers generate a similar series of MS3 product ions each with 2 m/z lower. Hence, the most prominent MS3 product ion for EO is at 379.2 (red arrows), while for DHO, the most prominent signature MS3 product ion was at m/z 381.2.

Enlarged parts of each product ion scan are shown in **Fig S2, A-D**. In each case the blue arrow identifies the DHO reference ion at m/z 381.2 (off scale). The red arrow identifies the molecular ion at 379.2 which is a signature ion for EO and each of the two isomers noted. **Panel E** shows the full product ion scan for samples derived from animals given icv Ang II and FAD288. Following HPLC separation, fraction 40 (isomer 2) was observed to generate abundant MS3 product ions (red arrows) whose intensity exceeded the DHO reference (Blue arrows). While all the molecular product ions characteristic of EO and isomer 1 were also present in this fraction, the most prominent molecular ion for isomer 2 was at 417.2 (green arrow) and not at m/z 379.2. **Panel F** gives and explicit comparison of the intensity of the signature product ion at m/z 379.2 relative to the DHO reference at m/z 381.2 for fractions 35 (EO) and 40 (isomer 2). This shows the very large amount of isomer 2 of EO in the animals that received icv Ang II with FAD286.

**Discussion**

Typically, MS3 spectra are especially challenging from a quantitative standpoint, especially when sample amounts are minuscule. To provide quantitation of EO and its two isomers using the MS3 approach it was critical to employ an external standard and a number of other operational modifications. Deuterated ouabain was considered as an internal plasma standard but was unsuitable because, in the amounts required for MS, it interferes with the ouabain RIA, and one of our goals was to investigate the relationship between EO immunoreactivity and the MS3 product ions. Instead, the close structural analog, exogenous DHO was chosen as an internal standard that could be added after SPE for three reasons. First, product molecular ions for endogenous DHO were not detectable in any of our rodent samples. Second, exogenous DHO has a mass to charge ratio that is a convenient 2 mass units greater than EO. Third, by using the appropriate isolation windows, product ions for EO and exogenous DHO could be observed simultaneously within the same ion trap cycles. This provided a considerable advantage over conventional MS3 approaches that typically alternate multi-ion isolation and fragmentation and thereby allowed for a more robust analysis. In addition, because these studies were performed offline from the LC step, it was possible to use long acquisition times that allowed for extensive averaging of the steady-state product ion intensities. By these means, it was possible to obtain quantitative information with an overall variance of ~20-25%.

**Structure-activity Relationships for EO isomers**

A number of deductions can be made about the general structures of the isomers from the MS spectra. First, both isomers are sugar-steroid conjugates that, by definition, have a mass-to-charge ratio (m/z) identical to EO, and the same elemental composition of C_29_H_44_O_12_. The distinct chromatographic polarities show that the two isomers are structurally distinct from EO, and from each other. Differences in the orientation (i.e., α vs β) and/or location of two or more hydroxyl groups are likely, and all affected hydroxyl groups are in the steroid moiety; i.e., EO and all isomers generated MS2 product ions at 445.3 m/z (an m/z ratio identical to ouabagenin) prior to MS3. Similarly, the sugar at C3 in all cases was a deoxyhexose, and rearrangement of hydroxyl groups within the isomers thus excludes the sugar.

In the case of isomer 1, switching the orientation of the hydroxyl at position 1 from β to α would increase polarity without significantly affecting immunocrossreactivity and biological function.

For isomer 2, the above noted structure-activity relationships along with the weak immunocrossreactivity and the lack of hypertensinogenic activity point to significant differences in the position and/or orientation of key hydroxyl groups in the A/B ring area; i.e., the same region where FAD286 blocks 11 and 19 hydroxylation. Loss of hydroxyls at these key positions could explain the inability of isomer 2 to induce hypertension in this study and also in normal pregnancy where isomer 2 is physiologically elevated [28].

**Quantitative and Qualitative Analyses of EO and Isomers**

In addition to routine RIA, the present work employed HPLC-RIA and offline HPLC-MS3 to provide unambiguous and specific evidence that plasma EO is elevated by brain Ang II.

In addition to EO, two circulating isomers were detected by LC-MS3. Because of its low crossreactivity with the R7 antiserum, isomer 2 was below the detection limit for the RIA in the control, A, and A+E groups (**Figs. 3A** and **H**) whereas it was readily detected in all groups by LC-MS3 **(c.f., Figs. 3A, B, C** and **H** vs **Fig. 4A**). The two EO isomers had similar elution characteristics and produced product ion spectra with the same mass to charge ratios as their counterparts in female rats [28]. Whereas the MS3 product ion spectra for isomer 1 and EO were indistinguishable in all aspects (not shown), isomer 2 can be recognized by the additional prominent molecular ion at 417.2 m/z in its product ion spectra (**Fig. S2 E, F**).

**Figure Legends**

**Figure S1. Identification and quantitation of plasma EO and isomers by SPE offline LC-mass spectrometry (MS).**

HPLC fractions were screened offline for unique molecular MS3 product ions of EO at 379.2 m/z. Ion peaks corresponding to EO were found in fraction 35 in each study group. Additional and/or more prominent peaks corresponding to EO isomers were found in fractions 31 and 40.

**Figure S2. SPE offline LC-mass spectrometry (MS) fractions showing MS3 Spectra for EO and two isomers and the effects of CNS Ang II infusion.**

**A**: Zoomed spectra for LC fraction 30 (F30) showing exogenous DHO reference ions at m/z 381.2 (dashed blue arrow). There are no endogenous product ions at m/z 379.2 for EO or its isomers in this fraction.

**B**: Zoomed spectra for LC fraction 31 (F31) showing endogenous product ions at m/z 379.2 for isomer 1 (red arrow) and the exogenous DHO reference ions at m/z 381.2 (dashed blue arrow).

**C**: Zoomed spectra for LC fraction 35 (F35) showing endogenous product ions at m/z 379.2 for EO (red arrow) and the exogenous DHO reference ions at m/z 381.2 (dashed blue arrow).

**D**: Zoomed spectra for LC fraction 40 (F40) showing endogenous product ions at m/z 379.2 for Isomer 2 (red arrow) and the exogenous DHO reference ions at m/z 381.2 (dashed blue arrow).

**E**: Full scan MS3 product ion spectra from icv Ang II+FAD286 LC fraction 40 showing endogenous product ions (solid red arrows) and exogenous DHO reference ions (dashed blue arrows). The horizontal dotted arrow (green) indicates the dominant molecular ion at m/z 417.2 unique to isomer 2.

**F**: Zoomed MS3 product ion spectra rats infused with icv Ang II (left) and icv Ang II + FAD286 (right). Diagnostic molecular product ions for EO in fraction 35 (F35) at m/z 379.2 (red arrow) and for DHO at m/z 381.2 (blue arrow) and in fraction 40 (F40, isomer 2) are shown.
